# The Utility of the SCAS-C/P to Detect Specific Anxiety Disorders Among Clinically Anxious Children

**DOI:** 10.1037/pas0000700

**Published:** 2019-05-09

**Authors:** Tessa Reardon, Cathy Creswell, Kathryn J. Lester, Kristian Arendt, Judith Blatter-Meunier, Susan M. Bögels, Jonathan R. I. Coleman, Peter J. Cooper, Einar R. Heiervang, Chantal Herren, Sanne M. Hogendoorn, Jennifer L. Hudson, Robert Keers, Heidi J. Lyneham, Carla E. Marin, Maaike Nauta, Ronald M. Rapee, Susanna Roberts, Silvia Schneider, Wendy K. Silverman, Mikael Thastum, Kerstin Thirlwall, Gro Janne Wergeland, Thalia C. Eley

**Affiliations:** 1 School of Psychology and Clinical Language Sciences, University of Reading; 2 School of Psychology, University of Sussex, and Institute of Psychiatry, Psychology and Neuroscience, Social, Genetic and Developmental Psychiatry (SGDP) Centre, King’s College London; 3 Department of Psychology and Behavioural Sciences, Aarhus University; 4 Department of Psychology, University of Basel; 5 Research Institute Child Development and Education, University of Amsterdam, and UvA Minds, Amsterdam, the Netherlands; 6 Psychiatry, Psychology and Neuroscience, Social, Genetic and Developmental Psychiatry (SGDP) Centre, King’s College London; 7 School of Psychology and Clinical Language Sciences, University of Reading; 8 Institute of Clinical Medicine, University of Oslo, and Department of Mental Health and Addiction, Oslo University Hospital, Oslo, Norway; 9 Department of Forensic Psychiatry, University of Basel Psychiatric Clinics; 10 Department of Child and Adolescent Psychiatry/De Bascule, Academic Medical Centre, Amsterdam, the Netherlands; 11 Centre for Emotional Health, Department of Psychology, Macquarie University; 12 Institute of Psychiatry, Psychology and Neuroscience, Social, Genetic and Developmental Psychiatry (SGDP) Centre, King’s College London, and School of Biological and Chemical Sciences, Queen Mary University of London; 13 Centre for Emotional Health, Department of Psychology, Macquarie University; 14 Child Study Center, Yale University School of Medicine; 15 Department of Clinical Psychology and Experimental Psychopathology, University of Groningen; 16 Centre for Emotional Health, Department of Psychology, Macquarie University; 17 Institute of Psychiatry, Psychology and Neuroscience, Department of Psychology, King’s College London; 18 Mental Health Research and Treatment Center (MHRTC), Ruhr University Bochum; 19 Child Study Center, Yale University School of Medicine; 20 Department of Psychology and Behavioural Sciences, Aarhus University; 21 School of Psychology and Clinical Language Sciences, University of Reading; 22 Anxiety Disorders Research Network and Division of Psychiatry, Department of Child and Adolescent Psychiatry, Haukeland University Hospital, Bergen, Norway; 23 Institute of Psychiatry, Psychology and Neuroscience, Social, Genetic and Developmental Psychiatry (SGDP) Centre, King’s College London

**Keywords:** child, mother, father, diagnosis, anxiety disorders

## Abstract

Questionnaire measures offer a time and cost-effective alternative to full diagnostic assessments for identifying and differentiating between potential anxiety disorders and are commonly used in clinical practice. Little is known, however, about the capacity of questionnaire measures to detect specific anxiety disorders in clinically anxious preadolescent children. This study aimed to establish the ability of the Spence Children’s Anxiety Scale (SCAS) subscales to identify children with specific anxiety disorders in a large clinic-referred sample (*N* = 1,438) of children aged 7 to 12 years. We examined the capacity of the Separation Anxiety, Social Phobia, Generalized Anxiety, and Physical Injury Fears (phobias) subscales to discriminate between children with and without the target disorder. We also identified optimal cutoff scores on subscales for accurate identification of children with the corresponding disorder, and examined the contribution of child, mother, and father reports. The Separation Anxiety subscale was able to accurately identify children with separation anxiety disorder, and this was replicated across all 3 reporters. Mother- and father-reported Social Phobia subscales also accurately identified children with social anxiety disorder, although child report was only able to accurately detect social anxiety disorder in girls. Using 2 or more reporters improved the sensitivity of the Separation Anxiety and Social Phobia subscales but reduced specificity. The Generalized Anxiety and Physical Injury Fears subscales failed to accurately identify children with the corresponding disorders. These findings have implications for the potential use of mother-, father-, and child-report SCAS subscales to detect specific disorders in preadolescent children in clinical settings.

## Public Significance Statement

We evaluated the ability of the Spence Children’s Anxiety Scale subscales to identify specific anxiety disorders in clinically anxious children aged 7 to 12 years. Findings provide support for the use of the Separation Anxiety and Social Phobia subscales to identify separation anxiety disorder and social anxiety disorder in clinical settings.

Anxiety disorders are among the most prevalent childhood mental health disorders (Polanczyk, Salum, Sugaya, Caye, & Rohde, 2015) and are associated with significant functional impairment and negative outcomes later in life (Bittner et al., 2007; Woodward & Fergusson, 2001). Anxiety disorders in children often co-occur (Waite & Creswell, 2014), and different anxiety disorders share some common features, including excessive anxiety or worry, physiological symptoms, and avoidance of anxiety-provoking situations or associated distress. Accurate identification of anxiety disorders and differentiation between different diagnoses is reliant on the availability of evidence-based assessment tools. Structured diagnostic interviews, such as the Anxiety Disorders Interview Schedule Child and Parent Interviews (ADIS-C/P; Silverman & Albano, 1996), are considered to be the gold-standard tool for identifying the presence of specific anxiety disorders in children. However, the ADIS-C/P is time consuming to complete, taking an average of 134 min when children are clinically anxious (Lyneham & Rapee, 2005), and requires clinical expertise to administer. Self-report questionnaire measures designed to detect elevated anxiety symptoms offer a time- and cost-effective alternative, and are therefore commonly used in clinical practice, both to identify specific anxiety disorders and to monitor response to treatment (Law & Wolpert, 2014).

The Spence Children’s Anxiety Scale (SCAS; Spence, 1998) is one widely used questionnaire measure designed to assess anxiety symptoms corresponding to the *Diagnostic and Statistical Manual of Mental Disorders* (4th ed., *DSM–IV*; American Psychiatric Association, 1994) anxiety disorders, with child- (SCAS-C) and parent-report (SCAS-P) versions available (hereafter, “SCAS-C/P”). The SCAS-C/P comprises subscales to assess the following *DSM–IV* anxiety disorders: separation anxiety, social phobia, generalized anxiety disorder, obsessive–compulsive problems, panic/agoraphobia, and physical injury fears (phobias). A large body of evidence has evaluated the psychometric properties of the SCAS-C/P, providing strong support for its reliability and validity. In particular, SCAS-C/P scores have good internal consistency (Orgilés, Fernández-Martínez, Guillén-Riquelme, Espada, & Essau, 2016) and test–retest reliability (Arendt, Hougaard, & Thastum, 2014). SCAS-C/P scores correlate more strongly with measures of internalizing symptoms (e.g., Strengths and Difficulties Questionnaire-Internalizing scale; Child Behavior Checklist-Internalizing subscale) than measures of externalizing symptoms (e.g., Strengths and Difficulties Questionnaire-Externalizing scale; Child Behavior Checklist-Externalizing subscale; Arendt et al., 2014; Nauta et al., 2004), indicating convergent and divergent validity. Discriminant validity is also illustrated in significantly higher SCAS-C/P scores among clinical than community samples (Arendt et al., 2014; Nauta et al., 2004; Spence, Barrett, & Turner, 2003; Whiteside & Brown, 2008).

Far fewer studies, however, have specifically examined the capacity of the SCAS-C/P to accurately identify children with anxiety disorders (sensitivity) and children without anxiety disorders (specificity), or the capacity of its subscales to identify children with and without specific anxiety disorders. As such, evidence relating to optimal cutoff scores on the SCAS-C/P and its subscales for accurate identification of anxiety disorders is also limited. Preliminary evidence has been reported for optimal cutoff scores on the SCAS-C/P for discriminating between a community sample and clinic-referred sample of children with anxiety disorders (Reardon, Spence, Hesse, Shakir, & Creswell, 2018).

Brown-Jacobsen, Wallace, and Whiteside (2011) reported sensitivity/specificity values associated with the SCAS-C/P subscales in a small sample of children and adolescents (*N* = 88; age = 7–18 years) but used predetermined cutoff scores based on normative data. Olofsdotter, Sonnby, Vadlin, Furmark, and Nilsson (2016) also examined the capacity of the SCAS-C/P subscales to identify specific anxiety disorders and reported data relating to alternative cutoff scores, but the sample included only adolescents (*N* = 104; 12–18 years). Evans, Thirlwall, Cooper, and Creswell (2017) provided evidence relating to the capacity of the SCAS subscales to identify recovery from specific anxiety disorders (*N* = 337, 7–12 years), and Whiteside, Gryczkowski, Biggs, Fagen, and Owusu (2012) specifically examined the capacity of the Obsessive–Compulsive subscale to identify children and adolescents with obsessive–compulsive disorders (clinical sample, *n* = 196, 7–18 years; community sample, *n* = 421, 8–13 years). However, the ability of the SCAS-C/P subscales to detect specific anxiety disorders in preadolescent children has not been established, nor are optimal subscale cutoff scores available for this population. The clinical characteristics of preadolescent children with anxiety disorders differ from adolescents with anxiety disorders (Waite & Creswell, 2014), and normative data (available on www.scaswebsite.com) indicates that SCAS-C/P scores also vary with age. It is therefore likely that optimal subscale cutoff scores will differ for preadolescent children and adolescents.

A multiple informant approach is widely recommended in the assessment of child mental health disorders (Achenbach, McConaughy, & Howell, 1987; Wren, Bridge, & Birmaher, 2004), and parent- and child-report anxiety questionnaires are both commonly used in clinical settings. Moderate levels of parent–child agreement are typically reported for SCAS-C/P scores (Arendt et al., 2014; Whiteside & Brown, 2008), and Arendt et al. (2014) also reported moderate mother–father agreement on SCAS-P scores. Limited agreement among reporters on the SCAS indicates that each reporter may provide unique information, and combining reporters may help improve the capacity of the SCAS to identify children with specific anxiety disorders. However, the benefit of combining child-, mother-, and father-reported SCAS subscales, and the optimal combination of reporters for accurate identification of children with specific anxiety disorders, are not yet established.

The aim of this study was to investigate the capacity of the SCAS-C/P subscales to detect specific anxiety disorders within a large clinic-referred sample (*N* = 1,438) of preadolescent children (aged 7–12 years). Specifically, we aimed to (a) establish the ability of each SCAS-C/P subscale to discriminate between children with and without that corresponding anxiety disorder as determined using the ADIS-C/P; (b) identify the optimal cutoff scores on the SCAS-C/P subscales to accurately identify the corresponding anxiety disorders; and (c) examine the relative contribution of child, mother, and father reports and the optimal combination of reporters to accurately identify specific disorders. SCAS data are available for mothers, fathers, and children in this study, allowing the accuracy of all three informants to be examined.

## Method

### Participants

Participants were children (aged 7–12 years) with a primary anxiety disorder and their mothers and fathers. The sample was recruited as part of the large multisite Genes for Treatment study (see Hudson et al., 2015, for further details). Inclusion criteria for the current sample were as follows: (a) the child was aged 7–12 years, (b) child- (SCAS-C) and mother-report (SCAS-P) data were available, and (c) the child had a primary anxiety disorder diagnosis consistent with the fifth edition of the *DSM* (*DSM–5*; American Psychiatric Association, 2013). At the time of the assessment, diagnoses were assigned according to *DSM–IV* (American Psychiatric Association, 1994) criteria, but to be consistent with *DSM–5*, children with a primary diagnosis of obsessive–compulsive disorder or posttraumatic stress disorder were excluded, and children with a primary diagnosis of selective mutism were included. Full sample details are provided in Table 1. The sample included 1,438 children (50.5% female) recruited across eight sites; father-report data (SCAS-P) were available for 953 children. The most common primary diagnoses were generalized anxiety disorder (42.4%), social anxiety disorder (22.2%), separation anxiety disorder (21.4%), and specific phobia (11.4%), with a mean Clinical Severity Rating (CSR) for primary diagnoses of 6.17 (*SD* = 1.0). Across diagnostic profiles, anxiety diagnoses included generalized anxiety disorder (75.0%), social anxiety disorder (63.5%), separation anxiety disorder (51.7%), specific phobia (49.7%), panic disorder with/without agoraphobia (2.2%), agoraphobia with/without panic disorder (1.6%), selective mutism (1.3%), and anxiety disorder not otherwise specified (1.3%). Nonanxiety diagnoses included attention-deficit hyperactivity disorder (11.0%), oppositional defiant disorder (10.6%), major depressive disorder/dysthymia (9.5%), and obsessive–compulsive disorder (5.9%).[Table-anchor tbl1]

Differences between children with father-report data available (*n* = 953) and those without father-report data (*n* = 485) were examined. There were no significant differences between the two subsamples on gender (χ^2^ = .47, *p* = .50), age, *t*(1006) = .85, *p* = .40, the SCAS-C/P total or subscale scores (*p* = .18–.99), or the presence of social anxiety disorder (62.9% vs. 64.7%, χ^2^ = .46, *p* = .50). There were significant differences between children with and without father-report data on the presence of separation anxiety disorder (48.7% vs. 57.5%, χ^2^ = 9.93, *p* = .002), generalized anxiety disorder (77.8% vs. 69.5%, χ^2^ = 11.96, *p* = .001), and specific phobias (57.6% vs. 46.0%, χ^2^ = 15.64, *p* < .001), but these differences reflected negligible effect sizes (Cramer’s V = .08–.11).

### Procedure

Data collected as part of the pretreatment assessment in the Genes for Treatment study was used in the current study (see Hudson et al., 2015 for further details). Children (*N* = 1,438) completed the SCAS-C, and mothers (*n* = 1,438) and fathers (*n* = 953) completed the SCAS-P. The ADIS-C/P was used to assign anxiety and comorbid diagnoses, and associated CSRs in all sites except at Bochum, where the Diagnostisches Interview bei psychischen Störungen im Kindes- und Jugendalter (Kinder-DIPS) [Schneider, Unnewehr, & Margraf, 2009] was used.^1^ All trials were approved by site-specific research ethics committees. Parents provided consent, and children provided assent.

### Measures

#### Spence Children’s Anxiety Scale Child and Parent Versions

The SCAS consists of corresponding child (SCAS-C; Spence, 1998) and parent (SCAS-P; Nauta et al., 2004) report questionnaires designed to assess symptoms of *DSM–IV–TR* (American Psychiatric Association, 1994) anxiety disorders. Each questionnaire includes 38 items rated on a 4-point scale (0 to 3; *never* to *always*), and the SCAS-C includes six additional positive filler items. The SCAS-C/P comprise six subscales addressing Separation Anxiety (six items), Generalized Anxiety (six items), Social Phobia (six items), Obsessive–Compulsive Behaviors (six items), Panic and Agoraphobia (nine items), and Physical Injury Fears (five items), and yields a total score (sum of responses to 38 items) and subscale scores (sum of responses to items on each subscale). In cases with missing data (<25% missing items), total and subscale scores reflect the average for completed items. Evaluation studies have provided strong support for the six-factor structure (Orgilés et al., 2016) and psychometric properties of the SCAS-C/P (e.g., Arendt et al., 2014; DeSousa et al., 2014; Nauta et al., 2004; Spence et al., 2003). The internal consistency in the current sample was good to excellent (SCAS-C, α = .91; SCAS-P mother report, α = .88; SCAS-P father report, α = .88).

#### Anxiety Disorders Interview Schedule

Diagnostic status was assessed using the ADIS-C/P-IV (Silverman & Albano, 1996) across all sites, with the exception of Bochum, where the German equivalent, Kinder-DIPS (Schneider, Unnewehr, & Margraf, 2009) was used. The ADIS-C/P consists of independent parent and child interviews, and its reliability and validity are widely reported (Silverman, Saavedra, & Pina, 2001). The presence and severity of anxiety disorders were assessed across all sites; and all sites (with the exception of Bergen) also assessed comorbid mood and externalizing disorders with this interview. Diagnoses were assigned if a child met the *DSM–IV* (American Psychiatric Association, 1994) criteria and received a CSR of 4 or above, based on the composite parent and child report (see Hudson et al., 2015, for further details). As per interview schedule guidance, when there were discrepancies between the child and parent reports, diagnoses were assigned if symptoms were reported by either the child or the parent, and the higher CSR was assigned as the overall CSR. Good interrater reliability (κ ≥ 0.8) for clinician-assigned diagnoses within samples used in this study are reported elsewhere (Creswell, Apetroaia, Murray, & Cooper, 2013; Hudson et al., 2014; Lyneham, Abbott, & Rapee, 2007).

### Data Analytic Approach

The ability of four SCAS-C/P subscales (Separation Anxiety, Social Phobia, Generalized Anxiety, Physical Injury Fears) to identify corresponding *DSM–5* (American Psychiatric Association, 2013) anxiety disorders (separation anxiety disorder; social anxiety disorder; generalized anxiety disorder; specific phobia) was examined. There were not sufficient panic disorder (2.2%) or agoraphobia (1.6%) diagnoses to examine the functioning of the Panic/Agoraphobia subscale.

Analyses examining only child- and/or mother-report SCAS subscale scores included the total sample (*N* = 1,438), and the subsample for which father report was available (*n* = 953) was used for analyses that included father-report SCAS subscale scores.

There are different published norms and *t* scores for preadolescent girls and boys for the SCAS (available on www.scaswebsite.com), and therefore it is likely that optimal subscale cutoff scores designed to detect the corresponding disorders will similarly vary for girls and boys. To determine whether it was appropriate to consider girls and boys separately in subsequent analyses, first, gender differences on these four SCAS-C/P subscale scores (independent samples *t* tests) were examined for each reporter (child, mother, father; see Online Supplement 1 of the online supplemental materials). Significant gender differences (*p* < .05) were observed on all child-report subscales, three mother-report subscales (Separation Anxiety, Generalized Anxiety, Physical Injury Fears), and the father-report Separation Anxiety subscale. To allow a consistent approach across analyses, girls and boys were considered separately in all subsequent analyses.

The capacity of each of the four SCAS-C/P subscales (based on child, mother, and father reports) to discriminate between children with and without the related anxiety disorder was examined using (a) independent sample *t* tests (and Cohen’s *d*), and (b) receiver operating characteristics (ROCs). ROC analyses produce an area under the curve (AUC) statistic, ranging from 1.0 (indicating perfect classification of children with/without the disorder) to .50 (indicating chance-level classification of children with/without the disorder). In line with previous studies using ROC analyses to examine child anxiety measures (van Gastel & Ferdinand, 2008; Villabø, Gere, Torgersen, March, & Kendall, 2012), a minimum threshold of an AUC of .70 was used to indicate that the SCAS-C/P subscale was at least moderately accurate in identifying the corresponding anxiety disorder. In cases in which the AUC was >.70, the sensitivity (correct classification of children with the target anxiety disorder) and specificity (correct classification of children without the target anxiety disorder) values for alternative cutoff scores were also examined. Identifying optimal cutoff scores involves a trade-off between sensitivity and specificity. With a focus on identifying the target disorder (and not missing cases), sensitivity was prioritized, and the optimal cutoff score reflected the score with sensitivity >.80 and specificity >.70. If it was not possible to achieve this .80/.70 combination, cutoff scores with lower sensitivity values (<.80) and specificity values >.60 were considered. For optimal cutoff scores, overall correct classification (i.e., number and percentage correctly classified) was also calculated.

Agreement between child–mother, child–father, and mother–father report on the four subscale scores was examined using Pearson correlations. Four logistic regressions were then used to examine the unique contribution of child, mother, and father reports in identifying the four target anxiety disorders (separation anxiety disorder, social anxiety disorder, generalized anxiety disorder, specific phobia). For each regression model, the corresponding child-mother-father subscale scores were entered using the block-entry method. In cases in which the ROC analyses indicated that the SCAS-C/P subscale was at least moderately accurate at identifying the corresponding anxiety disorder (i.e., AUC >.70), and an optimal cutoff score was identified, the sensitivity/specificity associated with each combination of reporters was also examined. It is possible to combine information from multiple reporters in different ways. In keeping with the standard approach used to combine information from multiple reporters in diagnostic interviews, and with the aim of maximizing the capacity to identify specific disorders, an “OR-rule” was used (i.e., children who scored above the cutoff score for at least one reporter were classed as “above the cutoff” overall). For each combination of reporters (child–mother, child–father, mother–father, child-mother-father), the following were calculated: (a) the proportion of children with the target anxiety disorder who scored above the optimal cutoff score on the corresponding subscale for at least *one* reporter (sensitivity), and (b) the proportion of children without the target anxiety disorder who scored below the optimal cutoff score on the corresponding subscale for *each* reporter (specificity). The total number (and percentage) of children who were correctly classified was also calculated, that is, children with the target anxiety disorder who scored above the optimal cutoff score on the corresponding subscale for at least one reporter plus children without the target disorder who scored below the optimal cutoff score on the corresponding subscale for each reporter.

## Results

### Discriminating Between Children With and Without Specific Anxiety Disorders

Differences on SCAS-C/P subscales among children with and without the target anxiety disorder are displayed in Table 2. Mean SCAS-C/P subscale scores were significantly higher among children with the target disorder than those without the target disorder, and this finding was replicated across reporters (child, mother, father) and gender groups. Differences between children with and without the target disorder were large across reporters for the Separation Anxiety subscale (*d* = .82–1.31) and small across reporters for the Generalized Anxiety subscale (*d* = .26–.42). Corresponding differences on the Social Phobia subscale ranged from large for mother report (*d* = .84–1.02), to medium-large for father report (*d* = .72–.96), and medium (*d* = .55–.77) for child report. Differences between children with and without specific phobias ranged from medium for the mother and father Physical Injury Fears subscale (*d* = .52–.72) to small for the corresponding child subscale (*d* = .41–.43).[Table-anchor tbl2]

### ROC Analyses

ROC analyses for each SCAS-C/P subscale for the three reporters (child, mother, and father) are displayed in Table 3. The Separation Anxiety subscale (child, mother, and father reports) was able to accurately identify separation anxiety disorders among both girls and boys (AUC = .73–.82). Optimal cutoff scores for each reporter were associated with sensitivity values >.70 (.70–.78) and corresponding specificity values >.60 (.62–.75).[Table-anchor tbl3]

The mother- and father-report Social Phobia subscale was able to accurately identify social anxiety disorders among both girls and boys (AUC = .70–.77). Optimal cutoff scores for mother and father reports were associated with sensitivity values of .70 to .71 among girls and .66 to .67 among boys, with corresponding specificity values of .69 to .71 among girls and .63 to .67 among boys. The child-report Social Phobia subscale achieved an AUC >.70 among girls (AUC = .71) but not boys (AUC = .65). Among girls, the optimal cutoff score on the child-report Social Phobia subscale achieved sensitivity of .67 and specificity of .65.

The Generalized Anxiety subscale was not able to accurately identify children with generalized anxiety disorder (AUC < .70 for child, mother, and father reports). The Physical Injury Fears subscale also failed to identify children with specific phobias (AUC < .70) for child or mother report. The father-reported Physical Injury Fears subscale, however, did achieve an AUC of .70 among girls (but not boys), and the associated optimal cutoff score achieved sensitivity and specificity values of .61 and .71, respectively.

### Using Multiple Informants

Correlations between child–mother, child–father, and mother–father report on the four subscales are displayed in Online Supplement 2 of the online supplemental materials. Across all subscale and gender groups, mother–father agreement ranged from .43 to .71, child–mother agreement ranged from .35 to .55, and child–father agreement from .26 to .51. Mother–father correlation coefficients ranged from .67 to .70 for the Separation Anxiety subscale to .43 to .48 for the Generalized Anxiety subscale. Child–mother correlation coefficients were similar on the Separation Anxiety and Physical Injury Fears subscales (.50–.55), and ranged from .35 to .42 for the Social Phobia and Generalized Anxiety subscales. Child-father correlation coefficients ranged from .44 to .51 on the Separation Anxiety and Physical Injury Fears subscales, to .26 to .29 on the Social Phobia and Generalized Anxiety subscales.

Table 4 displays findings from logistic regressions examining the contribution of child, mother, and father reports in identifying separation anxiety disorder, social anxiety disorder, generalized anxiety disorder, and specific phobias. Higher scores on the Separation Anxiety subscale for each reporter were associated with separation anxiety disorder among girls and boys (odds ratio [*OR*] = 1.11–1.27), indicating that each reporter made a unique contribution. The Nagelkerke and Cox and Snell *R*-squared statistics indicated that the separation anxiety model explained .35 to .46 of the variance among girls and .26 to .35 among boys.[Table-anchor tbl4]

Child-, mother-, and father-reported Social Phobia subscale scores also each made a significant contribution in identifying social anxiety disorders (*OR* = 1.10–1.18), and overall, the model explained .25 to .34 of the variance among girls and .18 to .24 among boys.

Higher scores on the Generalized Anxiety subscale were not, however, associated with generalized anxiety disorders based on child, mother, or father reports, and overall the generalized anxiety disorder model explained very little of the variance among girls or boys (Nagelkerke = .03/.02, Cox & Snell = .05/.04). Similarly, child-reported Physical Injury Fears subscale scores were not associated with specific phobias. Both father- and mother-reported Physical Injury Fears subscale scores each made a significant contribution to identifying specific phobias among girls (*OR*= 1.24 and 1.12, respectively); and mother report made a significant contribution to identifying specific phobias among boys (*OR* = 1.11). Overall, the specific phobia models, however, explained a small amount of the variance (girls, Nagelkerke = .12, Cox & Snell = .17; boys, Nagelkerke = .08, Cox & Snell = .11).

Sensitivity and specificity values associated with using two or more reporters were calculated for subscales when optimal cutoff scores were identified for each reporter (i.e., Separation Anxiety subscale and Social Phobia subscale among girls). As displayed in Table 5, combining two or three reporters improved the Separation Anxiety subscale sensitivity (.88–.92) but reduced its specificity (.44–.60). This reduction in specificity was less marked for mother–father report (specificity = .57–.60) than either mother–child (.50–.52), father–child (.49), or mother-father-child (.44–.45).[Table-anchor tbl5]

Similarly, combining two or three reporters improved the Social Phobia subscale’s sensitivity among girls (.87–.92) but reduced its specificity (.40-.56). Again, mother–father report produced higher specificity (.56) on the Social Phobia subscale than other reporter combinations.

## Discussion

We examined the capacity of the SCAS-C/P subscales to detect specific anxiety disorders (separation anxiety disorder, social anxiety disorder, generalized anxiety disorder, specific phobias) within a large, multisite, clinically anxious sample (*N* = 1,438) of children aged 7 to 12 years. There was variation in the extent to which scores on each subscale were able to discriminate between children with and without that corresponding anxiety disorder and the accuracy with which each subscale identified children with the target disorder.

The Separation Anxiety subscale score was able to discriminate between children with and without separation anxiety disorder, with significantly higher scores among children with than without separation anxiety disorder based on child, mother, and father reports (*d* = .82–1.31). This subscale also identified children with separation anxiety disorder with a moderate to good level of accuracy across the three reporters (AUC = .73–.82), and the optimal cutoff scores achieved an acceptable sensitivity/specificity balance (>.70/>.60). The Separation Anxiety subscale’s ability to accurately identify separation anxiety disorders in preadolescent children is in line with previous illustrations of its ability to accurately identify recovery from the corresponding anxiety disorder within the same age group (Evans et al., 2017) and its stronger predictive capacity than other SCAS subscales among adolescents (Olofsdotter et al., 2016).

The performance of the Social Phobia subscale showed some variation across reporters. The mother- and father-report Social Phobia subscale score discriminated between children with and without social anxiety disorders, with significantly higher scores among the former (*d* = .72–1.02), and also identified children with social anxiety disorder with a moderate level of accuracy (AUC = .70–.77). The optimal cutoff scores on the mother- and father-report Social Phobia subscale achieved acceptable sensitivity/specificity (for girls, .70–.71/.69–.71; for boys, .66–.67/.63–.67). Interestingly, these positive findings in relation to the parent-report Social Phobia subscale contrast with previous findings that the Social Phobia subscale failed to accurately identify recovery from social anxiety disorders (Evans et al., 2017). The parent-report Social Phobia subscale’s utility as an identification tool may therefore be greater than its utility to monitor treatment response. Similar to mother and father reports, the child-report Social Phobia subscale scores were also significantly higher among children with than without social anxiety disorder (girls, *d* = .77; boys, *d* = .55). Child report, however, only identified social anxiety disorder with an acceptable level of accuracy among girls (AUC = .71), with sensitivity/specificity values of .67/.65. Previous studies that include adolescents report positive findings in relation to the Social Phobia subscale’s ability to identify social anxiety disorders using both self-report and parent report (Brown-Jacobsen et al., 2011; Olofsdotter et al., 2016). The limited capacity of the social anxiety SCAS-C items to discriminate between a clinically anxious and community sample of preadolescent children is, however, reported elsewhere (Reardon et al., 2018). It is therefore possible that preadolescent children have limited ability to differentiate between developmentally appropriate and clinically significant social anxieties, but this ability improves with age.

The capacity of the Generalized Anxiety subscale score to discriminate between children with and without generalized anxiety disorder was limited. Although child-, mother-, and father-reported Generalized Anxiety subscale scores were significantly higher among children with than without generalized anxiety disorder, effect sizes were small (*d* = .26–.42). The Generalized Anxiety subscale also failed to accurately identify children with generalized anxiety disorder across reporters (AUC <.70). Doubt surrounding the Generalized Anxiety subscale’s ability to accurately detect generalized anxiety disorder is also reported elsewhere. Brown-Jacobsen et al. (2011) reported poorer performance for the Generalized Anxiety subscale compared with other subscales in relation to the sensitivity/specificity achieved in a sample of children and adolescents, and Nauta et al. (2004) reported similarly high scores on the parent-report Generalized Anxiety subscale among children with generalized anxiety disorder as those with other anxiety disorders. Interestingly the predictive capacity of the MASC Generalized Anxiety subscale is similarly limited (Villabø et al., 2012). There are, however, also more positive illustrations of the capacity of both the SCAS and the RCADS (a derivative of the SCAS) Generalized Anxiety subscales to detect generalized anxiety disorder (Chorpita, Moffitt, & Gray, 2005; Ebesutani et al., 2010; Olofsdotter et al., 2016), but as these studies include adolescents, it is possible that the SCAS and the RCADS Generalized Anxiety subscales are better able to detect generalized anxiety disorder in adolescents than preadolescent children and that the ability to identify clinically significant levels of worry improves with age.

Given that the SCAS Generalized Anxiety subscale addresses anxiety symptoms that are common across anxiety disorders (general worry, worries about bad things happening, physical symptoms), it may not be surprising that its capacity to discriminate between children with generalized anxiety disorder and children with other anxiety disorders is limited. Indeed, although studies examining the factor structure of the SCAS provide support for a six-correlated-factor model, corresponding to the six subscales (Orgilés et al., 2016), an alternative model with five correlated factors and a higher order Generalized Anxiety factor has also been proposed (Nauta et al., 2004), suggesting the Generalized Anxiety subscale is assessing an underlying general anxiety trait that is common across disorders. In order to develop a measure that can specifically detect generalized anxiety disorder, it may be necessary to adopt a bifactor approach and examine the capacity of individual items or a subset of items that can detect variance unique to generalized anxiety disorder, after the common variance (or general anxiety) across disorders is accounted for. Moreover, studies examining the reliability of the ADIS-C/P report lower interrater reliability for generalized anxiety disorder diagnoses compared with other anxiety diagnoses (Lyneham et al., 2007). Generalized anxiety disorder may be considered a less coherently defined construct than disorders that are characterized by specific or situational fears, and thus potentially harder to detect, particularly among young children. Further work is therefore needed to determine how best to maximize accurate identification of generalized anxiety disorders specifically among preadolescent children.

The capacity of the Physical Injury Fears subscale to identify specific phobias was also limited. Child-, mother-, and father-reported Physical Injury Fears subscale scores were each significantly higher among children with than without specific phobias, with medium effect sizes for mother and father reports (*d* = .52–.72) but small effect sizes for child report (*d* = .41–.43). Both the mother- and child-report Physical Injury Fears subscale, however, failed to accurately identify children with specific phobias (AUC < .70), and the father-report Physical Injury subscale identified only children with specific phobias with an acceptable level of accuracy among girls (AUC = .70), with sensitivity/specificity of .61/.71. The failure of the SCAS Physical Injury Fears subscale to accurately identify children with specific phobias is consistent with other illustrations of its limited discriminatory capacity (Brown-Jacobsen et al., 2011; Nauta et al., 2004). Studies also indicate that internal consistency is lower for the SCAS Physical Injury Fears subscale (Arendt et al., 2014) and Phobia subscales on other anxiety questionnaires (Muris, Mannens, Peters, & Meesters, 2017) compared with other subscales. Indeed, as each item on the Physical Injury Fears subscale addresses a different specific fear (e.g., fear of dogs, fear of the dark, fear of doctors/dentists), it may not be surprising that summing the score across these items does not discriminate between children with and without specific phobias. Rather than focusing on the frequency of different fears, questionnaire measures may need to assess the presence of specific fears and assess the level of impairment associated with any specific fear in order to accurately detect the presence of a specific phobia.

### Using Multiple Informants

This study illustrated that child, mother, and father reports each made a significant unique contribution in identifying children with separation anxiety disorder and social anxiety disorder, and using multiple reporters improved the sensitivity of the Separation Anxiety and Social Phobia subscales. As such, if the priority is to identify children with these disorders, and not miss cases, it may be beneficial to use more than one reporter (and increase the subscales’ sensitivity to >.84). Perhaps unsurprisingly, using two or more reporters did, however, reduce the subscales’ specificity. Therefore, although using a second reporter can help identify some children who would otherwise be missed, this is at the expense of an increase in false positives. This reduction in specificity was less marked for mother–father report than alternative reporter combinations, suggesting that mother–father report may be the optimal combination of reporters for the Separation Anxiety and Social Phobia subscales. Given that child–mother and child–father agreement was low on these subscales, it is not surprising that combining child and parent report introduced more false positives than relying on the closely related mother and father reports. Moreover, diagnoses based on the ADIS-C/P are more strongly associated with parent report than child report among preadolescent children (e.g., Evans et al., 2017). The dominant influence of parent report on diagnostic outcomes may therefore partly account for the stronger predictive capacity of parent-report questionnaires compared with child-report questionnaires. Collecting information from two parents is of course not always practical or feasible, and in these cases, using one parent report (either mother or father) can still identify children with separation anxiety disorder or social anxiety disorder with an acceptable level of accuracy (sensitivity = .66−.78). It is also important to note that we focused on identifying specific anxiety disorders, and consequently, we prioritized sensitivity to identify optimal cutoff scores, and we explored one approach to combining information from multiple reporters (i.e., children who scored above the cutoff for at least one reporter were classed as “above the cutoff” overall). However, if the priority was to identify children *without* specific anxiety disorders or to “rule out” specific disorders, it would be useful to consider alternative cutoff scores (e.g., prioritize specificity) and alternative approaches to combining information from multiple reporters (e.g., only children who score above the subscale cutoff for all reporters are classed as “above the cutoff” overall).

Mother–father agreement was only moderate on the Generalized Anxiety subscale, perhaps because the Generalized Anxiety items address internalizing processes (rather than observable behaviors), and, as noted previously, may address a less coherent construct than other subscales. Nevertheless, given the failure of the Generalized Anxiety subscale score to discriminate between children with and without generalized anxiety disorder across reporters, it is not surprising that no individual reporter made a significant unique contribution to identifying children with generalized anxiety disorder. When information from each reporter on the Physical Injury subscale was combined, only father report made a small significant unique contribution in identifying girls with specific phobias, and only mother report made a small significant unique contribution in identifying boys with specific phobias. These differences in the accuracy of mother and father reports on the Physical Injury Fears subscale, together with differences in optimal cutoff scores identified for mother and father reports on the Separation Anxiety and Social Phobia subscales, further highlight the importance of examining mother and father reports separately when considering a multi-informant approach to assessing child anxiety disorders.

### Implications

This study has implications for the potential use of the SCAS-C/P subscales to detect specific anxiety disorders in preadolescent children in clinical practice. Findings provide support for the use of the child- and parent-report Separation Anxiety subscale for identifying children with separation anxiety disorders. Findings also support the use of the parent-report Social Phobia subscale for identifying children with social anxiety disorders, and the child-report Social Phobia subscale for identifying girls with social anxiety disorders. This study provides data relating to both mother and father optimal cutoff scores, and so offers potential for application in settings in which only mother or father report is available. When multiple reporters are available, clinicians and researchers will need to weigh the improved capacity to identify the presence of separation anxiety disorder and social anxiety disorder associated with using multiple reporters against the reduced capacity to correctly identify the absence of separation anxiety disorder and social anxiety disorder. These findings are of particular importance to clinical settings in which questionnaire measures are commonly used as a time- and cost-effective means to identify potential diagnoses. Moreover, the RCADS (Chorpita, Yim, Moffitt, Umemoto, & Francis, 2000) is a derivative of the SCAS, and items on the SCAS Separation Anxiety and Social Phobia subscales also appear on the RCADS. These findings therefore have relevance to clinical services that routinely use the RCADS when it would be possible to use the SCAS Separation Anxiety and Social Phobia subscales items to identify children with the corresponding disorders. Importantly, the study suggests that the SCAS Generalized Anxiety subscale and Physical Injury Fears subscale should not be relied upon as measures to specifically identify children with generalized anxiety disorder and specific phobias in clinical populations.

### Limitations

There are a few limitations associated with this study. We examined the capacity of SCAS-C/P subscales to detect four anxiety disorders, but there were not a sufficient number of children with either panic disorder or agoraphobia to examine the capacity of the Panic/Agoraphobia subscale to detect children with these disorders. Standard diagnostic interview schedules were used to assess diagnoses, but it was not possible to evaluate interrater reliability for clinician-assigned diagnoses across all sites included in the sample. Generalized anxiety disorder was the most common diagnosis within the sample, and the fact that only a relatively small proportion of children (25%) did not have generalized anxiety disorder may have contributed to the SCAS subscale’s failure to accurately detect this disorder. Moreover, all children in this sample met criteria for at least one anxiety disorder, and therefore we were not able to examine the capacity of the SCAS subscales to discriminate between children with specific anxiety disorders and children without any anxiety disorders. Indeed, the variance on SCAS subscale scores was limited in this study, and it is likely that our results underestimate the capacity of the subscales to detect the target disorders compared with what we may expect to find in a community sample. Similarly, this study examined how well the SCAS can identify specific anxiety disorders within clinical populations, but we were not able to examine its capacity of to discriminate between children with and without any anxiety disorders.

It is also important to acknowledge that the SCAS was designed to assess symptoms consistent with *DSM–IV* (American Psychiatric Association, 1994) anxiety disorders. The SCAS items addressing obsessive and compulsive behaviors are therefore not consistent with the *DSM–5* (American Psychiatric Association, 2013) classification of anxiety disorders, in which obsessive–compulsive disorder is no longer classified as an anxiety disorder; and no SCAS item(s) specifically address the newly classified anxiety disorder, selective mutism. Changes from *DSM–IV* to *DSM–5* in the diagnostic criteria for anxiety disorders were, however, minor and do not alter the relevance of the other SCAS items and subscales. It will nevertheless be important for future research to examine the capacity of the SCAS (or subset of items) to specifically detect children with selective mutism. Indeed, Muris et al. (2017) report the close association between the SCAS Social Anxiety subscale and a new Selective Mutism scale, suggesting the capacity of the SCAS Social Anxiety subscale to detect children with selective mutism warrants investigation.

This study provides support for the ability of the SCAS Separation Anxiety and Social Phobia subscales to identify preadolescent children with separation anxiety disorder and social anxiety disorder in clinical populations, and provides optimal cutoff scores for mother, father, and child reports. It will also be important for future research to evaluate the capacity of mother-, father-, and child-report SCAS subscales to detect specific anxiety disorders among adolescents.

## Supplementary Material

10.1037/pas0000700.supp

## Figures and Tables

**Table 1 tbl1:** Sample Characteristics

Characteristic	*n* (%) or *M* (*SD*)
Total Sample (*NI*)	1,438
Site, *n* (%)	
Sydney, Australia	748 (52.0)
Reading, UK	400 (27.8)
Bergen, Norway	111 (7.7)
Aarhus, Denmark	66 (4.6)
Bochum, Germany	55 (3.8)
Oxford, UK	29 (2.0)
Miami, Florida, USA	27 (1.9)
Amsterdam, the Netherlands	2 (.1)
Age (years), *M* (*SD*)	9.89 (1.70)
Range	7–12
Gender	
Female, *n* (%)	726 (50.5)
SES, *n* (%)	
Higher/professional^a^	600 (41.7)
Other employed	323 (22.5)
Unemployed	38 (2.6)
Missing	467 (32.5)
SCAS-C (child report; *N* = 1,438), *M* (*SD*)	
Total score	36.11 (17.6)
Separation Anxiety subscale	7.01 (4.1)
Social Phobia subscale	6.04 (3.9)
Generalized Anxiety subscale	7.51 (3.8)
Physical Injury Fears subscale	4.47 (2.8)
SCAS-P (mother report; *N* = 1,438), *M* (*SD*)	
Total score	36.47 (14.5)
Separation Anxiety subscale	8.23 (4.1)
Social Phobia subscale	8.21 (4.0)
Generalized Anxiety subscale	7.54 (3.2)
Physical Injury Fears subscale	4.67 (2.9)
SCAS-P (father report; *N* = 953), *M* (*SD*)	
Total score	31.18 (13.2)
Separation Anxiety subscale	6.92 (3.9)
Social Phobia subscale	7.27 (3.8)
Generalized Anxiety subscale	6.27 (2.9)
Physical Injury Fears subscale	4.30 (2.7)
ADIS-C/P primary diagnosis, *n* (%)	
Separation anxiety disorder	308 (21.4)
Social anxiety disorder	319 (22.2)
Generalized anxiety disorder	609 (42.4)
Panic disorder/agoraphobia	22 (1.5)
Specific phobia	164 (11.4)
Selective mutism	3 (.2)
Anxiety disorder NOS	13 (.9)
Primary diagnosis CSR, *M* (*SD*)	6.17 (1.0)
Presence of anxiety disorder, *n* (%)	
Separation anxiety disorder	743 (51.7)
Social anxiety disorder	913 (63.5)
Generalized anxiety disorder	1,078 (75.0)
Panic disorder	31(2.2)
Agoraphobia	23 (1.6)
Specific phobia	715 (49.7)
Selective mutism	18 (1.3)
Anxiety disorder NOS	18 (1.3)
Presence of other psychiatric disorders,^b^ *n* (%)	
OCD	85 (5.9)
Major depressive disorder or dysthymia	137 (9.5)
ADHD	158 (11.0)
ODD	152 (10.6)
*Note*. SES = socioeconomic status; SCAS-C = Spence Children’s Anxiety Scale - Child Version; SCAS-P = Spence Children’s Anxiety Scale - Parent Version; ADIS-C/P = Anxiety Disorder Interview Schedule - Child and Parent Interviews; Anxiety disorder NOS = anxiety disorder not otherwise specified; Panic disorder = panic disorder with or without agoraphobia; Agoraphobia = agoraphobia with or without panic disorder; CSR = Clinical Severity Rating; OCD = obsessive–compulsive disorder; ADHD = attention-deficit hyperactivity disorder; ODD = oppositional defiant disorder.	
^a^ Higher/professional = managers, directors, senior officials, professional occupations. ^b^ Other psychiatric disorders >1%.	

**Table 2 tbl2:** Differences in Child, Mother, and Father Reports on SCAS-C/P Subscales Among Children With and Without the Target Anxiety Disorder

SCAS-C/P subscale	Reporter	Gender	Target disorder *M* (*SD*)	No target disorder *M* (*SD*)	*t* test (Cohen’s *d*)
Separation anxiety	Child	Girls	9.28 (3.76)	5.61 (3.55)	*t*(724) = 13.37*** (*d* = 1.00)
			(*n* = 411)	(*n* = 315)	
		Boys	7.94 (3.93)	4.90 (3.46)	*t*(709) = 10.95*** (*d* = .82)
			(*n* = 332)	(*n* = 379)	
	Mother	Girls	10.55 (3.33)	6.17 (3.34)	*t*(724) = 17.56*** (*d* = 1.31)
			(*n* = 411)	(*n* = 315)	
		Boys	10.03 (3.62)	5.85 (3.43)	*t*(709) = 15.80*** (*d* = 1.19)
			(*n* = 332)	(*n* = 379)	
	Father	Girls	9.03 (3.53)	5.27 (3.12)	*t*(473) = 12.21*** (*d* = 1.13)
			(*n* = 252)	(*n* = 223)	
		Boys	8.40 (3.84)	5.14 (3.20)	*t*(475) = 10.09*** (*d* = .92)
			(*n* = 212)	(*n* = 265)	
Social phobia	Child	Girls	7.48 (3.90)	4.71 (3.27)	*t*(724) = 9.80*** (*d* = .77)
			(*n* = 458)	(*n* = 268)	
		Boys	6.34 (3.90)	4.36 (3.33)	*t*(709) = 6.84*** (*d* = .55)
			(*n* = 455)	(*n* = 256)	
	Mother	Girls	9.73 (3.81)	5.97 (3.56)	*t*(724) = 13.15*** (*d* = 1.02)
			(*n* = 458)	(*n* = 268)	
		Boys	9.16 (3.74)	6.13 (3.45)	*t*(709) = 10.69*** (*d* = .84)
			(*n* = 455)	(*n* = 256)	
	Father	Girls	8.54 (3.73)	5.21 (3.18)	*t*(473) = 9.93*** (*d* = .96)
			(*n* = 297)	(*n* = 178)	
		Boys	8.19 (3.67)	5.64 (3.27)	*t*(475) = 7.50*** (*d* = .72)
			(*n* = 302)	(*n* = 175)	
Generalized anxiety	Child	Girls	8.47 (3.93)	6.93 (3.35)	*t*(724) = 4.82*** (*d* = .42)
			(*n* = 535)	(*n* = 191)	
		Boys	7.19 (3.55)	6.16 (3.57)	*t*(709) = 3.30*** (*d* = .29)
			(*n* = 543)	(*n* = 168)	
	Mother	Girls	8.10 (3.31)	6.93 (3.32)	*t*(724) = 4.18*** (*d* = .36)
			(*n* = 535)	(*n* = 191)	
		Boys	7.51 (3.17)	6.59 (2.83)	*t*(709) = 3.36*** (*d* = .31)
			(*n* = 543)	(*n* = 168)	
	Father	Girls	6.55 (2.89)	5.80 (2.83)	*t*(473) = 2.39* (*d* = .26)
			(*n* = 365)	(*n* = 110)	
		Boys	6.33 (2.84)	5.56 (2.76)	*t*(475) = 2.43* (*d* = .27)
			(*n* = 376)	(*n* = 101)	
Physical injury fears	Child	Girls	5.46 (2.65)	4.29 (2.82)	*t*(671) = 5.54*** (*d* = .43)
			(*n* = 381)	(*n* = 292	
		Boys	4.45 (3.08)	3.31 (2.48)	*t*(652) = 5.18*** (*d* = .41)
			(*n* = 334)	(*n* = 320)	
	Mother	Girls	5.74 (2.90)	3.99 (2.52)	*t*(671) = 8.21*** (*d* = .64)
			(*n* = 381)	(*n* = 292)	
		Boys	5.12 (2.82)	3.55 (2.63)	*t*(652) = 7.35*** (*d* = .58)
			(*n* = 334)	(*n* = 320)	
	Father	Girls	5.15 (2.57)	3.36 (2.40)	*t*(450) = 7.45*** (*d* = .72)
			(*n* = 271)	(*n* = 181)	
		Boys	4.71 (2.72)	3.34 (2.57)	*t*(447) = 5.44*** (*d* = .52)
			(*n* = 278)	(*n* = 201)	
*Note.* SCAS-C/P = Spence Children’s Anxiety Scale-Child and Parent Versions.					
* *p* < .05. ** *p* < .01. *** *p* < .001.					

**Table 3 tbl3:** Receiver Operating Characteristics (ROCs) for Child-, Mother-, and Father-Report SCAS-C/P Subscales

SCAS-C/P subscale	ROC statistics	Girls			Boys		
		Child	Mother	Father	Child	Mother	Father
Separation anxiety	SEP (positive), *n*	411	411	252	332	332	212
	No SEP (negative), *n*	315	315	223	379	379	265
	AUC	.76	.82	.79	.73	.80	.74
	Optimal cut score	6.5	8.5	6.5	5.5	7.5	6.5
	Sensitivity	.75	.73	.78	.73	.78	.70
	Specificity	.64	.75	.70	.62	.69	.69
	Correct classification, *n* (%)	507 (70.0)	537 (74.0)	354 (74.5)	477 (67.1)	521 (73.3)	331 (69.4)
Social phobia	SAD (positive), *n*	458	458	297	455	455	302
	No SAD (negative), *n*	268	268	178	256	256	175
	AUC	.71	.77	.75	.65	.72	.70
	Optimal cut score	5.5	7.5	6.5		7.5	6.5
	Sensitivity	.67	.71	.70		.66	.67
	Specificity	.65	.71	.69		.67	.63
	Correct classification, *n* (%)	481 (66.3)	515 (71.0)	329 (69.3)		470 (66.1)	312 (65.4)
Generalized anxiety	GAD (positive), *n*	535	535	365	543	543	376
	No GAD (negative), *n*	191	191	110	168	168	101
	AUC	.61	.62	.58	.58	.57	.58
	Optimal cut score						
	Sensitivity						
	Specificity						
	Correct classification, *n* (%)						
Physical injury fears	SP (positive), *n*	381	381	271	334	334	248
	No SP (negative), *n*	292	292	181	320	320	201
	AUC	.62	.68	.70	.60	.67	.65
	Optimal cut score			4.5			
	Sensitivity			.61			
	Specificity			.71			
	Correct classification, *n* (%)			292 (64.6)			
*Note*. Correct classification = true positives + true negatives. SCAS-C/P = Spence Children’s Anxiety Scale - Child and Parent Versions; SEP = separation anxiety disorder; AUC = Area Under the Curve; SAD = social anxiety disorder; GAD = generalized anxiety disorder; SP = specific phobia.							

**Table 4 tbl4:** Contribution of Child-, Mother-, and Father-Reported SCAS-C/P Subscales in Identifying Children With the Target Anxiety Disorders

Target anxiety disorder	Girls				Boys			
	*b* (Wald)	Odds ratio [95% CI]	*R*^2^	Model	*b* (Wald)	Odds ratio [95% CI]	*R*^2^	Model
SEP								
Constant	−3.89 (103.29***)				−3.10 (97.98***)			
Child	.13 (12.69***)	1.14 [1.06, 1.22]	.35 (Cox & Snell) .46 (Nagelkerke)	χ^2^(3) = 201.15***	.14 (18.14***)	1.14 [1.08, 1.22]	.26 (Cox & Snell) .35 (Nagelkerke)	χ^2^(3) = 144.63***
Mother	.24 (31.99***)	1.27 [1.17, 1.39]			.16 (16.32***)	1.17 [1.09, 1.27]		
Father	.14 (11.70***)	1.15 [1.06, 1.25]			.11 (7.62**)	1.11 [1.03, 1.20]		
SAD								
Constant	−2.50 (62.37***)				−1.82 (39.59***)			
Child	.12 (12.37***)	1.12 [1.05, 1.20]	.25 (Cox & Snell) .34 (Nagelkerke)	χ^2^(3) = 137.19***	.11 (11.08***)	1.11 [1.05, 1.18]	.18 (Cox & Snell) .24 (Nagelkerke)	χ^2^(3) = 93.04***
Mother	.15 (16.78***)	1.16 [1.08, 1.25]			.15 (17.56***)	1.16 [1.08, 1.24]		
Father	.17 (18.94***)	1.18 [1.10, 1.27]			.10 (6.89**)	1.10 [1.03, 1.18]		
GAD								
Constant	.00 (00, *p* = 1.00)				.21 (.38, *p* = .54)			
Child	.07 (3.92, *p* = .05)	1.07 [1.00, 1.14]	.03 (Cox & Snell) .05 (Nagelkerke)	χ^2^(3) = 14.54**	.04 (1.33, *p* = .25)	1.04 [.97, 1.12]	.02 (Cox & Snell) .04 (Nagelkerke)	χ^2^(3) = 11.36***
Mother	.05 (1.59, *p* = .21)	1.05 [.97, 1.14)			.07 (2.60, *p* = .11)	1.08 [.99, 1.17]		
Father	.05 (1.05, *p* = .31)	1.05 [.96, .1.15]			.05 (1.19, *p* = .28)	1.05 [.96, 1.16]		
SP								
Constant	−1.09 (19.70***)				−.88 (17.96***)			
Child	.01 (.10, *p* = .75)	1.01 [.93, 1.11]	.12 (Cox & Snell) .17 (Nagelkerke)	χ^2^(3) = 59.65***	.07 (2.88, *p* = .09)	1.07 [.99, 1.16]	.08 (Cox & Snell) .11 (Nagelkerke)	χ^2^(3) = 38.97***
Mother	.11 (4.84*)	1.12 [1.01, 1.23]			.11 (4.42*)	1.11 [1.01, 1.23]		
Father	.21 (15.32***)	1.24 [1.11, 1.37]			.09 (2.74, *p* = .10)	1.09 [.98, 1.21]		
*Note*. SCAS-C/P = Spence Children’s Anxiety Scale-Child and Parent Versions; CI = confidence interval; SEP = separation anxiety disorder; SAD = social anxiety disorder; GAD = generalized anxiety disorder; SP = specific phobia.								
* *p* < .05. ** *p* < .01. *** *p* < .001.								

**Table 5 tbl5:** Identifying Separation Anxiety Disorders and Social Anxiety Disorders Using the Corresponding SCAS-C/P Subscale With Multiple Reporters (Child, Mother, Father)

SCAS-C/P reporter(s)	Separation anxiety disorder						Social anxiety disorder		
	Girls			Boys			Girls		
	Sensitivity	Specificity	Correct classification *n* (%)	Sensitivity	Specificity	Correct classification *n* (%)	Sensitivity	Specificity	Correct classification *n* (%)
Child–mother	.88	.52	528 (72.7)	.92	.50	494 (69.5)	.87	.51	533 (73.4)
Child–father	.91	.49	339 (71.3)	.92	.49	325 (68.1)	.87	.46	341 (71.8)
Mother–father	.88	.60	355 (74.7)	.84	.57	329 (68.9)	.84	.56	348 (73.2)
Child-mother-father	.93	.45	335 (70.5)	.94	.44	317 (66.5)	.92	.40	343 (72.2)
*Note*. Sensitivity, specificity, and correct classification values calculated using optimal cutoff scores on child/mother/father report SCAS-C/P subscales reported in Table 3. Correct classification = true positives + true negatives. SCAS-C/P = Spence Children’s Anxiety Scale-Child and Parent Versions.									

## Footnotes

1 The main analyses outlined below were conducted separately for the total sample (*N* = 1,438) and the sample excluding sites where the Kinder-DIPS was used (*n* = 1,383). The results obtained from these two sets of analyses were consistent with each other, so only the results relating to the total sample are reported here.
