# Supplementary material for: The Utility of the SCAS-C/P to Detect Specific Anxiety Disorders Among Clinically Anxious Children
Source: Psychol Assess. 2019 May 9;31(8):1006–18. doi: 10.1037/pas0000700 (PMC6671872; doi:10.1037/pas0000700)
Supplement: Supplementary file 1 [file PAS-2018-2368_Suppl.docx]

Online Supplements

Online Supplement 1

Gender differences on child, mother and father reported SCAS subscales

| SCAS subscale | Reporter | Girls  Mean (SD) | Boys  Mean (SD) | *t* test (cohen’s *d*) |
| --- | --- | --- | --- | --- |
| Separation anxiety | child | 7.68 (4.09)  *n* = 726 | 6.32 (3.98)  *n* = 712 | *t*(1436) = 6.39*** (*d* = .34) |
|  | mother | 8.65 (3.98)  *n* = 726 | 7.80 (4.09)  *n* = 712 | *t*(1436) = 4.00*** (*d* = .21) |
|  | father | 7.27 (3.83)  *n* = 475 | 6.59 (3.85)  *n* = 478 | *t*(951) = 2.73** (*d* = .18) |
|  |  |  |  |  |
| Social phobia | child | 6.45 (3.91)  *n* = 726 | 5.63 (3.82)  *n* = 712 | *t*(1436) = 4.07*** (*d* = .21) |
|  | mother | 8.34 (4.13)  *n* = 726 | 8.07 (3.91)  *n* = 712 | *t*(1436) = 1.29, *p* = .20 |
|  | father | 7.29 (3.88)  *n* = 475 | 7.25 (3.78)  *n* = 478 | *t*(951) = .17, *p* =.87 |
| Generalised anxiety | child | 8.06 (3.85)  *n* = 726 | 6.94 (3.58)  *n* = 712 | *t*(1436) = 5.70*** (*d* = .30) |
|  | mother | 7.79 (3.35)  *n* = 726 | 7.29 (3.12)  *n* =712 | *t*(1436) = 2.90* (*d* = .15) |
|  | father | 6.37 (2.89)  *n* = 475 | 6.17 (2.84)  *n* = 478 | *t*(951) = 1.11, *p* = .27 |
| Physical injury fears | child | 4.98 (2.73)  *n* = 726 | 3.95 (2.86)  *n* = 712 | *t*(1436) = 6.98*** (*d* = .37) |
|  | mother | 4.96 (2.87)  *n* = 726 | 4.38 (2.84)  *n* = 712 | *t*(1436) = 3.85*** (*d* = .20) |
|  | father | 4.47 (2.67)  *n* = 475 | 4.13 (2.78)  *n* = 478 | *t*(951) = 1.91, *p* = .06 |

****p*<.001

***p*<.01

**p*<.05

Online Supplement 2

Correlations between child-mother, child-father and mother-father report on SCAS-C/P subscales

| SCAS subscale | Girls | | | Boys | | |
| --- | --- | --- | --- | --- | --- | --- |
|  | child-mother | child-father | mother-father | child-mother | child-father | mother-father |
| Separation anxiety | *r* = .55***  *n* = 726 | *r* = .51***  *n* = 475 | *r* = .67***  *n* = 475 | *r* = .51***  *n* = 712 | *r* = .44***  *n* = 478 | *r* = .70***  *n* = 478 |
|  |  |  |  |  |  |  |
| Social phobia | *r* = .42***  *n* = 726 | *r* = .29***  *n* = 475 | *r* = .62***  *n* = 475 | *r* = .35***  *n* = 712 | *r* = .26***  *n* = 478 | *r* = .61***  *n* = 478 |
|  |  |  |  |  |  |  |
| Generalised anxiety | *r* = .41***  *n* = 726 | *r* = .27***  *n* = 475 | *r* = .43***  *n* = 475 | *r* = .35***  *n* = 712 | *r* = .26***  *n* = 478 | *r* = .48***  *n* = 478 |
|  |  |  |  |  |  |  |
| Physical injury fears | *r* = .50***  *n* = 726 | *r* = .45***  *n* = 475 | *r* = .65***  *n* = 475 | *r* = .52***  *n* = 712 | *r* = .49***  *n* = 478 | *r* = .71***  *n* = 478 |

****p*<.001
